# Does Subjective Dietary Knowledge Affect Sugar-Sweetened Carbonated Beverages Consumption and Child Obesity? Empirical Evidence from the Inner Mongolia Autonomous Region in China

**DOI:** 10.3390/ijerph18073713

**Published:** 2021-04-02

**Authors:** Zeqi Liu, Wei Si, Qiran Zhao, Chang Tao

**Affiliations:** 1 College of Economics and Management, China Agricultural University, Beijing 100083, China; lzqtx@cau.edu.cn (Z.L.); siwei@cau.edu.cn (W.S.); zhaoqiran@cau.edu.cn (Q.Z.); 2 Academy of Global Food Economics and Policy, China Agricultural University, Beijing 100083, China

**Keywords:** children, overweight, obesity, sugar-sweetened carbonated beverages, dietary knowledge

## Abstract

Worldwide, overweight and obesity have become an important public health problem affecting the health of children and adolescents. In China, the prevalence of overweight and obesity has reached 19 percent among the 6–17-year-old age group. Although studies have shown that regular consumption of sugar-sweetened beverages (SSBs), especially sugar-sweetened carbonated beverages (SSCBs), is positively correlated with overweight and obesity among children, the research on ways to reduce SSBs consumption is scarce. This study fills this gap by analyzing data on nearly 4000 students aged between 9–15 from the Inner Mongolia Autonomous Region in China, exploring possible influential pathways between subjective dietary knowledge, SSCBs consumption, and child obesity. The estimation results show that SSCBs consumption significantly mediates the relationship between dietary knowledge and the incidence of overweight and obesity; the mediated effects are different among subgroups. Therefore, improving dietary knowledge related to the lowing of SSBs consumption to reduce the obesity risk may be considered a possible way to reduce the prevalence of overweight and obesity among children.

## 1. Introduction

Overweight and obesity have become an important public health problem affecting the health of children and adolescents worldwide [1]. These factors are associated with poorer health and wellbeing and higher health-care costs for both the present and future lifetime [2,3].

Between 1975 and 2016, the worldwide prevalence of overweight and obesity among children (5–19 years) more than quadrupled (from 4 percent to 18 percent) [4]; between 2000 and 2016, the prevalence of obesity in children (5–19 years) more than doubled (from 2.9 percent to 6.8 percent) [1]. Although the problem of overweight and obesity was once considered to only exist in high-income countries, it is now increasing in low- and middle-income countries. In the United States, the obesity prevalence among children aged 2–19 increased from 15.4 to 18.5 percent between 2005–2006 and 2015–2016 [5]. In Australia, the prevalence of overweight and obesity among children aged 2–17 has stayed relatively stable around 25 percent since 2007–2008 [6]. In the European World Health Organization member states, from 2007 to 2013, the prevalence of overweight and obesity among children aged 6–9 in more than half of the countries exceeded 25%, among which the prevalence rates in Greece, Italy, and Spain exceeded 40% [7]. Overweight and obesity among children have shown a concerning upward trend in Asia. It was estimated that in 2019, nearly half of the world’s children who were overweight or obese under 5 years of age lived in Asia [4], or 19.1 million. In China, the prevalence of overweight and obesity among children and adolescents almost tripled between 2002 and 2020. According to *The Chinese National Nutrition and Health Survey In 2002*, the prevalence was 6.6 percent for the 7–17 age group [8]; according to the “*Report on Nutrition and Chronic Disease Status of Chinese Residents (2020)*”, it has risen to 19 percent for the 6–17 aged group, reaching approximately 34 million (estimated by *China Population and Employment Statistics Yearbook-2019* data). Specifically, the obesity rate in the Inner Mongolia Autonomous Region (the surveyed region of the data used in this paper) ranks sixth in the nation, and the overweight rate ranks fourth, with values of 37.6% and 19.6%, respectively.

The fundamental cause of obesity is an energy imbalance between calories consumed and calories expended [4,9], which is often caused by a complex mixture of dietary [10,11,12,13], lifestyle [14,15], genetic [16], psychological [17,18], sociocultural [19,20], economic, and environmental factors [13,21]. The intake of energy-dense foods high in sugar is considered one of the main causes of human energy imbalance [4]. According to a series of systematic reviews [22,23,24,25], consumption of sugar-sweetened beverages (SSBs), particularly sugar-sweetened carbonated beverages (SSCBs), may be a key contributor to the epidemic of overweight and obesity in children and adults. In China, according to the “*China Children’s Sugary Beverage Consumption Report*” released in 2018, the average daily beverage consumption of urban children increased from 329 mL to 715 mL between 1998 and 2008 [26]. The 2010–2012 survey on the nutritional and health status of Chinese residents reported that 61.9 percent of children aged 6–17 drank beverages at least once a week. A 2014 national intervention program against obesity in Chinese children and adolescents aged 6–17 years reported that 66.6 percent of participants consumed SSBs and 9.6 percent consumed more than seven servings of SSBs per week [27]. Although there are differences in the methods and results obtained in these studies, they showed that children’s intake of free sugar from SSBs exceeded the upper limit recommended by WHO. WHO strongly recommends that the intake of free sugars obtained from food does not exceed 10% of total energy intake [28].

There is growing evidence that properly designed taxes on SSBs [29,30,31,32,33], especially if the aim is to increase retail prices by 20% or more [34], and specific interventions that can reduce added sugar intake [22,24] would result in a proportional reduction in SSBs consumption and thus affect the prevalence of overweight and obesity. In China, although studies have shown that regular consumption of sugar-sweetened beverages (SSBs), especially sugar-sweetened carbonated beverages (SSCBs), is positively correlated with overweight and obesity among Chinese children [27,35,36,37], the research on ways to reduce SSBs consumption to reduce the incidence of overweight and obesity is rare. Since a public opinion support environment for the taxation of sugary beverages has not yet been formed, if a tax on sugary drinks is rashly imposed, it will inevitably arouse public doubts.

To address this gap, this study aims to estimate the possible influence of dietary knowledge, SSCBs consumption, and child obesity in China. Drawing from the idea of “taking preventive measures” in Confucian culture, we determine whether students’ dietary knowledge will affect the consumption of SSCBs, thereby affecting the occurrence of overweight and obesity. If an influential pathway exists, it can provide a meaningful reference for the realization of obesity prevention and control goals for Chinese children; it can also promote the awareness of government and citizens with respect to the importance and necessity of a sugar-sweetened beverage tax.

To the best of our knowledge, the present study is the first to explore potential ways to reduce the incidence of overweight and obesity among Chinese children by reducing SSCBs consumption. Existing studies in China mainly focus on the positive correlation between SSCBs consumption and child obesity [27,35,36,37], but they have not continued to explore solutions in depth. To investigate the possible influential pathways, a field survey was designed to assess all sample students’ health status, subjective dietary knowledge, and consumption of SSCBs, and a simultaneous equation system was specified to perform mediation analysis by using three-stage least squares. This study aims to draw a general profile of the prevalence of overweight and obesity among children in the surveyed region and assess the possible ways to reduce this endemic prevalence. Through the analysis, it is also hoped that more targeted and cost-effective suggestions can be put forward to prevent further increases in the prevalence of overweight and obesity in Chinese children.

## 2. Materials and Methods

### 2.1. Materials

#### 2.1.1. Survey Design

The study consisted of a regionally representative sample of Inner Mongolia Autonomous Region children attending the fourth and fifth grades in elementary schools and of the seventh and eighth grades in junior high schools during the academic year 2019/2020. According to the *Monitoring Data on Chronic Diseases and Risk Factors (2017)* released by the Chinese Center for Disease Control and Prevention (CCDCP), the overweight and obesity rates in the Inner Mongolia Autonomous Region are higher than the national average. In the “Implementation Plan for Child and Adolescent Obesity Prevention and Control” formulated by the National Health Commission, the Ministry of Education, and four other departments of the People’s Republic of China, released in October 2020, this region was classified as a high-endemic area of overweight and obesity among children and adolescents in China [38]. The survey involved the following data collection modules:

- All students selected as survey subjects participated in physical measurements (i.e., height and weight) in their classrooms. Then, each completed a questionnaire, including personal characteristics (such as gender, age, hukou, sibling number, etc.), dietary knowledge items related to their cognition, and practical consumption of common snacks with a high sugar content.
- The caregiver of every student completed a questionnaire on parental information and the family socioeconomic status, such as parental age, education level, height and weight, and durable household assets, etc.

#### 2.1.2. Study Population

This study used a cross-sectional dataset collected in 2019, covering a respective sample of 36 compulsory schools, including 4105 children and their respective parents. Overall, the current analysis included:

- 36 schools from the Jining District, Ulanqab city (all compulsory education schools in this district), including 25 primary schools and 11 junior high schools.
- A randomly selected sample of 4105 students with valid data on physical measurements and semi-structured questionnaires, including 52.1 percent who were girls (Table 1). Among the entire sample, about 62.3 percent were primary school students, and 51.6 percent have rural hukou. [156 of sampled children (3.8 percent) did not complete the questionnaire for various reasons].
- 3949 family questionnaires (96.9 percent were completed by sampled parents, and the rest were completed by other relatives of the children).

#### 2.1.3. Sampling

The samples were collected by a multi-stage, stratified, random sampling procedure. In China, compulsory education is a nine-year education period and is divided into two stages. The first stage is the six-year elementary education. The second stage is the three-year junior high school education. Elementary schools and junior high schools in the surveyed region are separate, rather than nine-year uniform education schools. Since the project team plans to conduct a 3-year follow-up survey in this region of China, more classes were selected in the lower grades of each education stage to ensure that a large proportion of samples can be followed up. The specific steps were as follows:

- First, the project team obtained a list of compulsory schools (whether public or private schools) from Jining District’s bureau of education. Based on this, four project team numbers phoned the principal of each school to confirm the schedule, and 36 of the 37 compulsory education schools in the district participated in this survey.
- Then, the project team randomly chose two Grade 4 classes and one Grade 5 class in each primary school and randomly chose two Grade 7 classes and one Grade 8 class in each junior high school. A total of 104 classes (three of the schools had only one Grade 4 class that was the only choice; one of the schools did not have a Grade 5 class) were chosen.
- All students in selected classes were the research objects of the survey.

#### 2.1.4. Data Collection

To implement data collection, the project team prepared some necessary materials, such as investigator handbooks, portable scales, tape measures, student questionnaires, and family questionnaires. The questionnaires were developed based on similar surveys; for instance, China Health and Nutrition Survey, China Family Panel Studies, and previous surveys of this project team [13]. The project team evaluated the validity of the questionnaire through a small-scale pre-survey method and modified certain contents of the questionnaire based on the results of the survey, such as the type of sugar-sweetened beverages and the type of household fixed assets involved.

The present study was approved by the Ethics Committee of China Agricultural University. All procedures involving human participants followed the 1964 Declaration of Helsinki and its subsequent amendments. Before the fieldwork, all necessary permission from the local government, local education bureaus, and the sampled schools to conduct the survey was obtained. All students and their respective caregivers participating in this survey had full knowledge of the survey purpose and agreed with participation.

Physical measurements, including height and weight, were performed on every student by using a portable scale and tape measure. Then, each student filled out the modules of dietary knowledge items related to their cognition and the modules of practical consumption of SSBs in the questionnaire with the assistance of trained enumerators and teachers. After that, the children continued to complete other modules in the questionnaire in the classroom. The family questionnaire, involving family socioeconomic information, was taken home by the children and filled out by their respective caregiver, and then collected by the headteacher and given to us.

Of all 4105 students in the sample, 3949 completed the survey completely, involving all aspects and questionnaire information, including body measurement, dietary knowledge, carbonated beverage consumption table, and basic information of individuals and families. The information of these students constituted the database of statistical analysis and regression analysis, that is, the final sample included 3949 students.

### 2.2. Outcomes of Interests

#### 2.2.1. Body Mass Index–for–Age Z–Score, Overweight, and Obesity

In the physical measurement stage, the height (unit: m) and weight (unit: kg) of the students were measured. Then, their body mass index–for–age z–score (i.e., BMI–for–age z–score) was calculated by WHO AnthroPlus and used to assess students’ overall health status [39]. BMI–for–age z–score is a suitable growth reference for the 5–19-year age group and is recommended by the WHO for both clinical and epidemiological use [40,41]. The factors of sex and age are fully considered in the calculation process of the BMI–for–age z–score, irrespective of ethnicity [39]. The cut-off points for defining overweight and obesity are taken as +1 standard deviation(sd) and +2sd, coinciding with adult BMI at 25 kg/m^2^ and 30 kg/m^2^, respectively. Noting that, in this study, cut-off points for defining overweight are taken as BMI–for–age z–score > +1sd, including BMI–for–age z–score > +2sd, instead of the usual BMI–for–age z–score > +1sd but < +2sd; the cut-off point for defining obesity is taken as BMI–for–age z–score > +2sd. (Table 2 and Table 3).

#### 2.2.2. Dietary Knowledge

Dietary knowledge (DK) in this study is the degree of students’ subjective identification of a series of statements describing dietary knowledge. It is measured using a partial version of the *Diet Knowledge Table (DKT)* from *China Health* *and Nutrition Survey 2011-Child Questionnaires* [42]. The China Health and Nutrition Survey (CHNS), an ongoing open cohort, international collaborative project between the Carolina Population Center at the University of North Carolina at Chapel Hill and the National Institute for Nutrition and Health (NINH, former National Institute of Nutrition and Food Safety) at the Chinese Center for Disease Control and Prevention (CCDC), is a representative survey project that has lasted more than 30 years in China. The DKT consists of 12 items describing dietary knowledge. This survey had four items that are suitable for the cognitive ability of the sample group in this study, such as

- “Choosing a diet with a lot of fresh fruits and vegetables is good for one’s health”,
- “Eating a lot of sugar is good for one’s health”,
- “Consuming milk and dairy products is good for one’s health”,
- “The heavier one’s body is, the healthier he or she is”.

Since children may have difficulty understanding the six items that involve phrases such as “Choosing a diet high in fat is good for one’s health” and “Reducing the amount of fatty meat and animal fat in the diet is good for one’s health”, this article uses

- “The development of good eating habits is very beneficial to health”

As a representative. (Appendix A. Table A1)

The respondents were asked if he or she strongly agrees, somewhat agrees, neutral, somewhat disagrees, or strongly disagrees with each statement. The students were reminded that the question is not asking about their actual habits. The options were scored as strongly disagree = 1, disagree = 2, neutral = 3, agree = 4, strongly agree = 5, or unknown = 0. In the present study, DK is scored in the traditional method, in which the score for each statement is summed to a single total measure (Table 2).

#### 2.2.3. Sugar Intake from Sugar-Sweetened Carbonated Beverages (SSCBs)

Two indicators are used to measure sugar intake in sugar-sweetened carbonated beverages (SSCBs). Considering the limitations of the data research process, that is, the survey method used was to guide students to review the consumption and frequency of SSCBs in the past week, the recall data might be a rough estimate of the actual intake with relatively low accuracy. Therefore, two indicators are used for help in estimation and mutual verification. Before the formal investigation, a pilot survey was conducted. In particular, a forum with the school administrative staff and parent representatives was held to find out the possible choices of students, which helped to ensure the quality of the recovery data. During the investigation, the targeted students recorded their recall of SSBs consumption with possible help from trained investigators to ensure the quality of the recall data.

The first indicator is the consumption of sugar-sweetened carbonated beverages per day (units: milliliter per day; SSCBs_mL). Information about SSCBs consumption was obtained from the modules of practical consumption of SSBs. For every SSBs in the table, the number of milliliters per serving was defined, and one serving of SSCBs was defined as 350 mL. Students were asked how many servings they had consumed during the previous week. To facilitate students to understand the type and the capacity unit of SSCBs, the investigator provided images of SSCBs and provided unlabeled beverage bottles of different sizes as a reference during the investigation. Four levels were fixed for SSCBs consumption: (i) never or rarely per week, (ii) 1 serving per week, (iii) 2–3 servings per week, and (iv) at least 4 servings per week (noting the quantity). Then the indicator, SSCBs_mL, was calculated based on the number of SSCBs servings consumed by students per week and the quantity per serving. (Table 2)

To further provide complementary information on students’ SSCBs consumption, the present paper resorts to the second indicator, namely, *the consumption frequency of sugar-sweetened carbonated beverages (SSCBs_freq)*. Information about SSCBs_freq was also obtained from the modules of the practical consumption of SSBs, by asking each student how frequently they had drunk SSCBs throughout the previous week. Four levels were fixed for SSCBs consumption frequency: (i) never or rarely per week, (ii) 1 or 2 times per week, (iii) 3 to 5 times per week, and (iv) at least 7 times per week (noting the quantity) (Table 2).

#### 2.2.4. Control Variables

Based on the semi-structured questionnaires that student and their caregivers independently completed about their personal information and family socioeconomic status, a series of control variables (i.e., personal and family characteristics presented in Table 2) [43,44,45,46] were generated.

### 2.3. Estimation Method

**Figure 1:**
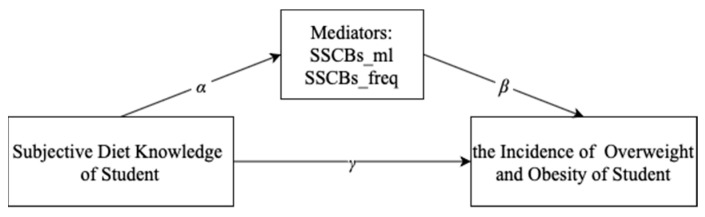
A hypothetical mediational model: Subjective dietary knowledge affects the incidence of overweight and obesity among students through SSCBs consumption.

Drawing from the idea of “taking preventive measures” in Confucian culture, the major objective of this study is to analyze the possible influential pathway of subjective dietary knowledge, SSCBs consumption, and child overweight and obesity, that is, the subjective dietary knowledge affects the incidence of overweight and obesity among students through SSCBs consumption. To meet the study’s objectives, a hypothetical mediational model is devised as shown in Figure 1, predicated on the following hypotheses:

**Hypothesis** **1.** *Subjective dietary knowledge will be related negatively to the consumption of sugar-sweetened carbonated beverages per day (SSCBs_mL).* ($\alpha_{1}<0$)

**Hypothesis** **2.** *Subjective dietary knowledge will be related negatively to the consumption frequency of sugar-sweetened carbonated beverages per week (SSCBs_freq).* ($\alpha_{2}<0$)

**Hypothesis** **3.** *SSCBs_mL will be related positively to the prevalence of overweight and obesity.* ($\beta_{1}>0$)

**Hypothesis** **4.** *SSCBs_freq will be related positively to the prevalence of overweight and obesity.* ($\beta_{2}>0$)

The simultaneous equation systems are specified as follows:

(1) $$\{\begin{array}{c} SSCBs\_ml_{i}=c+\alpha_{1}\cdot DK_{SI_{i}}+∆\cdot Controls_{i}+\mu_{ji}\\ Outcomes_{j\;i}=c+\beta_{1}\cdot SSCBs\_ml_{i}+\gamma \cdot DK_{i}+∆\cdot Controls_{i}+\varepsilon_{ji} \end{array}$$

(2) $$\{\begin{array}{c} SSCBs\_freq_{i}=c+\alpha_{2}\cdot DK_{SI_{i}}+∆\cdot Controls_{i}+\nu_{ji}\\ Outcomes_{j\;i}=c+\beta_{2}\cdot SSCBs\_freq_{i}+\gamma \cdot DK_{i}+∆\cdot Controls_{i}+\varepsilon_{ji} \end{array}$$

where the outcome variable, $Outcomes_{ji}$, is the *i*th student’s health status (i.e., BMI–for–age z–score, overweight, and obesity). $SSCBs\_ml_{i}$ is the consumption of sugar-sweetened carbonated beverages per day of the *i*th student. $SSCBs\_{\mathrm{freq}}_{i}$ is the consumption frequency of sugar-sweetened carbonated beverages per week of the *i*th student. The term α measures the correlation between subjective dietary knowledge and SSCBs consumption indicators; β measures the correlation between SSCBs consumption indicators and student’s health outcomes. The terms α and β are the coefficients of interest and will be used to calculate the indirect effects of the DK on students’ health status. The term γ measures the correlation between subjective dietary knowledge and student’s health outcomes, that is, the direct effect of DK on student’s health outcomes. The vector *X_i_* is comprised of a set of control variables (mentioned in Section 2.2.3), and ∆ is the related coefficient vector. The term c is the intercept, and ε and ν represent random errors that exist in a normal distribution. Here, *i* represents each of the observations. To gain a deeper understanding, the present paper analyzed heterogeneity among subgroups grouped according to personal or family characteristics, since these characteristics appeared to be effect modifiers or moderators.

The mediation analysis is performed using three-stage least squares (3SLS). For a multi-equation system, if the equation contains endogenous explanatory variables, the two-stage least squares (2SLS) estimation for each equation is consistent, but it is not the most efficient, as the single equation 2SLS ignores the possible existence of disturbance terms in different equations correlation. Since DK may be an endogenous explanatory variable affected by the control variables of individual and family characteristics, three-stage least squares (3SLS) [47] which is more efficient in this situation, are obtained to simultaneously estimate the simultaneous equation system. The three-stage least squares method of estimating a structural equation consists of three steps, the first of which serves to separate the exogenous part of the endogenous variable, the second to use the exogenous part for regression, and the third to obtain the estimation of the covariance matrix of the disturbance term of the entire system. Then, on this basis, generalized least squares estimation (similar to the seemingly unrelated regression estimation method [48]) is performed on the entire system.

Firstly, models of BMI–for–age z–score are analyzed starting with a model with SSCBs_mL as a mediator, followed by a model with SSCBs_freq as a mediator. All models exhibited a good fit for the data. The process is repeated for overweight, and then for obesity; both models exhibited good fits for the data, as shown in Table 4 and Table 5.

Then, mediation effects are identified by the standardized regression coefficients from 3SLS analysis. Generally, a given variable may be said to act as a mediator to the extent that it accounts for the relation between the dependent variable and the independent variable [49]. The mediating effect is generally calculated by the product-of-coefficients approach [50,51], which determines the mediation by multiplying the regression coefficients (α·β). It also needs to obtain the standard error of α·β, by dividing the product of the coefficients α and β by its standard error ($SE_{\alpha \beta }=\sqrt{\alpha^{2}\cdot SE_{\beta }^{2}+\beta^{2}\cdot SE_{\alpha }^{2}}$), to evaluate the statistical significance of the mediation effect. The coefficient γ represents the direct influence of DK on health outcome indicators. Complete mediation indicates that the mediator can fully explain the effects of the direct influence (do not reject the null hypothesis that γ=0), while partial mediation indicates the mediator can partially explain the direct influence (reject the null hypothesis that γ=0 at the characteristic significance level).

## 3. Results

### 3.1. Descriptive Analysis

#### 3.1.1. Descriptive Statistics of the Sample

Table 2 presents summary statistics of all of the variables used in the analysis. As presented in Table 2, the average BMI–for–age z–score was 0.33 among the entire sample. Almost one-third were overweight and obese, and more than half were obese. The overweight and obesity rate of children in the Inner Mongolia Autonomous Region is indeed at a high epidemic level.

The score of DK ranged from 1 to 25. A high score of DK indicated that students had a healthier cognition of the items related to dietary knowledge. The average score of subjective dietary knowledge (DK) was 20.7. Although students’ choices may not represent their actual habits, it can still be expected that students’ cognition and behavior would be consistent to a certain extent.

As for the consumption of SSCBs, 72.1 percent of the students drank SSCBs in the past week. Per capita and consumer SSCBs intake values were 38.1 (sd 46.1) and 52.8 (sd 46.5) mL/d, respectively. The average intake of boys was significantly higher than that of girls by 12.6 mL/d. Per capita and consumer SSCBs consumption frequencies were 1.3 (sd 1.6) and 1.8 (sd 1.6) servings per week, respectively. The average intake of boys was significantly higher than that of girls by 0.3 servings per week. Furthermore, the distribution of BMI among subgroups (grouped by individual and family characteristics) was analyzed and is presented in Section 3.1.2. Since these differences were observed without controlling for potential confounding factors, they can only be interpreted as suggestive. However, these observations still provide us with a useful reference for grouping in the heterogeneity analysis stage.

#### 3.1.2. The Distribution of BMI–for–Age Z–Score Among Subgroups

**Figure 2:**
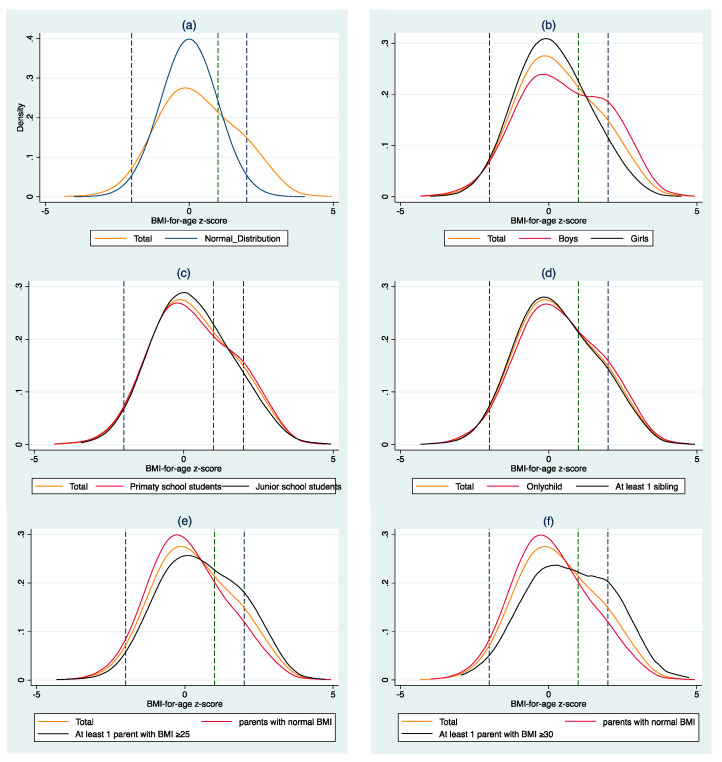
Distribution of body mass index-for-age z-score for entire sample, subgroups, and normal distribution (Mean = 0, Standard deviation = 1). (**a**): Distribution of BMI-for-age z-score for the entire sample and normal distribution; (**b**): Distribution of BMI-for-age z-score for the entire sample and subgroups (grouped by gender); (**c**): Distribution of BMI-for-age z-score for the entire sample and subgroups (grouped by school type); (**d**): Distribution of BMI-for-age z-score for the entire sample and subgroups (grouped by sibling number); (**e**,**f**): Distribution of BMI-for-age z-score for the entire sample and subgroups (grouped by parental health status).

Figure 2 presents the distribution of BMI–for–age z–score for entire sample and subgroups. As presented in Figure 2a, the overall distribution of students BMI-for-age was positioned to the right of the normal distribution, with a relatively flat right tail, which indicates that more were overweight and obese than the standard status. Figure 2b–f shows the distribution of BMI–for–age z–score among subgroups. Compared with the entire distribution, the distributions of both boys and girls were relatively flat, while girls’ distributions were slightly steeper than those of boys; the distribution of primary school students was relatively flat, while junior school students’ distribution was slightly steep; the distribution of only-child was relatively flat, while that of students with at least was sibling was slightly steep; the distribution of students whose father’s and mother’s BMIs were normal was slightly steep, that of students with at least one parent with BMI ≥ 25 was lightly flat, and that of students with at least one parent with BMI ≥ 30 was flatter.

Specifically for boys, the prevalence of overweight and obesity was 40 percent, among which the obesity rate was 25 percent. For girls, the two figures were 24 percent and 9 percent, respectively. For primary school students, the prevalence of overweight and obesity was 33 percent, among which the obesity rate was 18 percent; for junior school students, the two figures were 29 percent and 14 percent, respectively. For only-child, the prevalence of overweight and obesity was 34 percent, among which the obesity rate was 19 percent; for students with at least one sibling, the two figures were 30 percent and 15 percent, respectively. For students whose father’s and mother’s BMI were both normal (18.5 ≤ BMI_father < 25 and 18.5 ≤ BMI_mother < 25), the prevalence of overweight and obesity was 26 percent, among which the obesity rate was 11 percent; for students with at least one parent with BMI ≥ 25 (BMI_father ≥ 25 or BMI_mother ≥ 25), the two figures were 38 percent and 22 percent, respectively; for students with at least one parent with BMI ≥ 30 (BMI_father ≥ 30 or BMI_mother ≥ 30), the two figures were 44 percent and 28 percent, respectively. The prevalence of boys, junior school students, only-child, students having at least one parent with BMI ≥ 25, and students having at least one parent with BMI ≥ 30 was significantly higher than that of the compared subgroups (Table 3).

### 3.2. Systematic Analysis of a Hypothetical Mediational Model

As seen in Table 4 and Table 5, the results showed a significant negative relationship between DK and SSCBs consumption indicators (SSCBs_mL and SSCBs_freq) but a positive correlation between SSCBs consumption indicators and health outcomes (overweight and obesity). Specifically, the results in Table 4 suggest that for the model of overweight with SSCBs_mL as a mediator, for every 1-point gain in DK, the consumption of SSCBs per day significantly decreased by 0.806 mL/d (*p* < 0.01). Further, for every 1-mL/d gain in SSCBs_mL, the incidence of overweight increased by 0.002 sd (*p* < 0.05). For the model of obesity with SSCBs_mL as a mediator, a 1-point increase in DK was negatively associated with a 0.542 mL/d (*p* < 0.05) reduction in SSCBs_mL, while a 1-mL/d increase in SSCBs_mL was associated with a 0.004 sd increase in the obesity ratio (*p* < 0.01).

The results in Table 5 suggest that for the model of overweight with SSCBs_freq as a mediator, for every 1-point gain in DK, the consumption frequency of SSCBs per week significantly decreased by 0.04 drinks/w (*p* < 0.01). Further, for every 1-drink/w gain in SSCBs_freq, the incidence of overweight increased by 0.204 sd (*p* < 0.01). For the model of obesity with SSBCs_freq as a mediator, a 1-point increase in DK was negatively associated with a 0.35 drink/w (*p* < 0.05) reduction in SSCBs_ freq, while a 1-drink/w increase in SSCBs_freq was associated with a 0.136 sd increase in the obesity ratio (*p* < 0.01).

Table 4 presents the mediated effect of the indicator SSCBs consumption per day, while Table 5 presents that of the indicator SSCBs consum ption frequency per week. Both of the indicators mediated the relationship between subjective dietary knowledge and health outcomes, and SSCBs_freq had the stronger mediating effect. Specifically, with SSCBs_mL as a mediator, a 1-point increase in DK was associated with a 0.0019 sd decrease in the overweight ratio (*p* < 0.10) and a 0.0021 sd decrease in the obesity ratio (*p* < 0.05). With SSCBs_freq as a mediator, a 1-point increase in DK was associated with a 0.0044 sd (*p* < 0.01) and 0.0047 sd (*p* < 0.01) decrease in the overweight and obesity ratio, respectively.

### 3.3. Heterogeneous Effect of Mediation

The statistical analysis mentioned in Section 2.2.1 and Section 2.2.3 showed significant differences in the BMI–for–age z–score distribution, consumption of SSCBs, or consumption frequency of SSCBs among subgroups divided by personal/family characteristics (such as gender, school type, sibling number, and parental health status). Some personal and family characteristics appeared to be effective moderators. To gain a deeper understanding, the present paper analyzed the heterogeneity effect of mediation by gender subgroups. Table 6 presented the direct effects on the health outcomes (γ) and hypothetical mediators (α) and the effect of the mediator on the outcome (β) for the SSCBs consumption indicators for each subgroup. The mediated effect (α*β) for each indicator per subgroup was computed.

For the relationship of “Dietary knowledge with Overweight”, no significant mediated effect was found in subgroups of boys, junior school students, students whose father’s and mother’s BMI were normal (18.5 ≤ BMI_father < 25 and 18.5 ≤ BMI_mother < 25), and students with at least one parent with BMI ≥ 30 (BMI_father ≥ 30 or BMI_mother ≥ 30). Small but significant complete mediated effects were found in subgroups of girls, students with no sibling, and students with at least one parent with BMI ≥ 25 (BMI_father ≥ 25 or BMI_mother ≥ 25). Specifically, among girls or students with at least one parent with BMI ≥ 25, the complete mediated effect values of SSCBs_mL and SSCBs_freq were 0.004 sd (*p* < 0.05) and 0.005 sd (*p* < 0.01), respectively; among students with no sibling, the values were 0.004 sd (*p* < 0.05) and 0.005 sd (*p* < 0.05), respectively. Partial mediated effects were found in subgroups of primary school students and students with at least one sibling. Specifically, among primary school students, the partial mediated effect values of SSCBs_mL and SSCBs_freq were 0.008 sd (*p* < 0.01) and 0.007 sd (*p* < 0.01), respectively; among students with at least one sibling, the mediating effect value of SSCBs_freq was 0.003 sd (*p* < 0.05).

For the relationship of “Dietary knowledge with Obesity”, no significant mediated effect is found in subgroups of boys and students with no sibling. Small but significant complete mediated effects were found in subgroups of girls, primary school students, junior school students, and students with any type of parental health status. Specifically, among girls, the complete mediated effect values of SSCBs_mL and SSCBs_freq were 0.004 sd (*p* < 0.05) and 0.005 sd (*p* < 0.01), respectively; among primary school students and students with at least one parent with BMI ≥ 30, the complete mediating effect values of SSCBs_mL were 0.005 sd (*p* < 0.1) and 0.014 sd (*p* < 0.1), respectively; among junior school students and students whose father’s and mother’s BMI were normal, the complete mediating effect values of SSCBs_freq were 0.004 sd (*p* < 0.05) and 0.002 (*p* < 0.05), respectively; among students with at least one parent with BMI ≥ 25, the complete mediated effect values of SSCBs_mL and SSCBs_freq were 0.003 sd (*p* < 0.05) and 0.004 sd (*p* < 0.01), respectively. Partial mediated effects were found in subgroups of primary school students and students with at least one sibling, with SSCBs_freq as a mediator. Specifically, among primary school students, the partial mediated effect value of SSCBs_freq was 0.006 sd (*p* < 0.01); among students with at least one sibling, the mediating effect value of SSCBs_freq was 0.005 sd (*p* < 0.05).

## 4. Discussion

The prevalence of overweight and obesity among children and in the Inner Mongolia Autonomous Region is at a high level. As a high-endemic area of overweight and obesity among children and adolescents in China, the prevalence of overweight and obesity in the Inner Mongolia Autonomous Region is 13 percent higher than the national average (19 percent in the 6–17-year age group). In particular, the prevalence of overweight and obesity among boys in this region is twice the national average. This may represent the average level of twelve high-endemic areas of overweight and obesity among children in China, which has exceeded the overweight and obesity rates of children in some high-income countries, such as Australia [6]. The findings of this article provide the recent status of SSCBs consumption of a regionally representative sample of Inner Mongolian children and adolescents. Although the proportion of children in Inner Mongolia consuming SSCBs is relatively high (72.1 percent), the per capita intake is relatively low, 38.1 mL per day (sd 46.1) or 1.3 drinks per week (sd 1.6). Since the proportion of sugar per unit weight of SSCBs is 12 percent in China [52], by multiplying the average daily consumption of SSCBs by the proportion of added sugar in SSCBs, the per capita added-sugar intake derived from SSCBs was calculated as 4.6 grams per day. This finding is consistent with a previous study conducted in China on SSBs consumption among children and adolescents aged 6–17 years [27] but contradicts the findings of children in high-income countries. For example, in Australia, although the daily intake of added-sugar from SSCBs is nearly four times that of Chinese children in the present study, the proportion of children and adolescents who consumed SSCBs was less than 50 percent [53].

SSBs are a major contributor to children’s added sugar consumption. Although in clinical trials and epidemiological studies, evidence for an association between sugar consumption and weight gain is inconclusive [54], there is growing evidence that a reduction in SSBs consumption would result in a proportional decrease in the prevalence of overweight and obesity [22,24,29,30]. In China, studies have shown that regular consumption of SSBs, especially SSCBs, is positively correlated with overweight and obesity among children [27,35,36,37], but there are differences in the selection of obesity indicators. Some believe that SSBs consumption is independently associated with a high risk of abdominal obesity in children and adolescents, but not with general obesity [27]. Some reported that SSBs consumption is positively correlated with general obesity [36,37]; others reported that SSBs consumption is positively correlated with both abdominal obesity and obesity [35]. In the present study, there was a small but significant positive correlation between the consumption of SSCBs and the prevalence of both overweight and obesity. These correlation coefficients for girls were more significant and higher in absolute value than those of boys, although girls’ consumption and SSCBs consumption frequency were significantly lower than those of boys’. These correlation coefficients for primary school students were more significant and higher in absolute value than those of junior school students, which may be because most of the primary school students in the sample were younger than 12 (in childhood or early adolescence) when they participated in the survey. Compared with the relatively older junior high school students, they have different metabolic characteristics, and their BMI–for–age z–score is more likely to be impacted by SSCBs consumption.

Although taxing SSBs to improve population health outcomes has appeared as an effective intervention to address the rising prevalence of obesity in both high- and middle-income countries [29,30,31,32,33,34], there is no evidence that it can reduce the population overweight and obese risk permanently. Besides, the specific effects on tax fairness and health promotion are still being demonstrated, and a unified evaluation system to monitor the property and sustainability of taxes on SSBs is lacking. In China, the public opinion support environment for taxation of sugary beverages has not yet been formed, and the corresponding publicity and science education is still in its infancy. If a tax on sugary drinks is rashly imposed, it would inevitably arouse public doubts, and the consequences are unpredictable. In Chinese Confucianism, there is a concept of “taking preventive measures”, which means to prevent disasters before they happen. Some intervention experiments in children showed that behavioral intervention could reduce children’s sugar intake, but they had low compliance to dietary advice. Nutritional education, such as courses on general health issues and common advice on healthy diets, may not affect children’s behavior in the short term [23,55]. Another experiment to reduce children’s consumption of SSBs by instituting an educational course (for a period of six months) showed that primary school students are receptive to information on healthy diets, and adding educational programs to the primary school curriculum may help reduce the consumption of SSBs in the long term [56]. Since it is parents or caregivers, especially female roles, who decide what food to purchase for household consumption, their dietary knowledge might indirectly affect children’s behavior. However, although no studies have shown that female education has no effect on children’s nutrition, it cannot be assumed that there is a positive linear relationship between the two in some cases [57]. In China, although some studies evaluated the effects of comprehensive childhood obesity interventions on dietary intake among children [58], there is no relevant research on interventions in the consumption of SSCBs.

In the present paper, the estimation results of the hypothetical mediational models showed that the mediation effects of the two SSBC consumption indicators were small but significant. The results suggest that improving dietary knowledge related to the lowing of SSBs consumption to reduce the obesity risk may be considered a possible way to reduce the prevalence of overweight and obesity among children. These pathways of influence may have more obvious effects on younger children, since the mediation coefficients for primary school students were more significant and higher in absolute value than those for junior school students. This may be because compared with junior high school students, primary school students are still in the stage of cognitive formation, and their cognitive level and behavioral consistency are higher. Besides, the heterogeneity effect of mediation by gender subgroups indicated that the mediation coefficients for girls were more significant and higher in absolute value than those for boys. This may be because Chinese girls pay more attention to their appearance and diet compared with boys. Obesity prevention in children should be gender-focused, particularly for girls who reported SSCBs consumption but had a lower prevalence of obesity, even though more boys were overweight or obese than girls. Evidence with data from three large cohorts (i.e., Nurses’ Health Study, Health Professionals’ Follow-up Study, and Women’s Genome Health Study) suggests that regular consumption of SSBs exacerbates the genetic risk of obesity [24]. Moreover, as seen in this study, for students with at least one parent with BMI ≥ 30, it is no longer possible to influence the incidence of overweight and obesity by adjusting dietary knowledge and SSCBs consumption. Considering the individual differences in metabolic reactions to the intake of SSBs and the gender differences in the pathways of influence, the improvement of girls’ weight status and the accumulation of dietary knowledge may be passed on to the next generation, thereby permanently reducing the prevalence of obesity in the population.

To the best of our knowledge, the present study is the first to explore potential ways to reduce the incidence of overweight and obesity by reducing SSCBs consumption among 9–15 year-olds in China. The conclusions of this paper may enable Chinese policy-makers to design more targeted and cost-effective strategies and interventions to reduce SSBs intake of children and thus to prevent further increases in overweight and obesity prevalence. A more general implication is to lay the foundation for achieving zero growth in overweight and obesity among children and adolescents by providing a reference for policymakers to designate sugar consumption-intervention projects.

It is undeniable that there are some limitations to consider when evaluating the results of this article. First, although the structural equation model solves part of the endogeneity problem, a causal relationship still cannot be established due to the cross-sectional sample. Second, this study was limited to students aged 9–15, and the conclusions may not be applicable to other age groups. Expansion of sample coverage by designing reasonable random intervention experiments or tracking surveys to construct panel data might provide more reliable and generalizable results. Third, due to data limitations, the present paper did not involve students’ physical activity status in the analysis. Adding the physical activity module into the questionnaires does help draw more comprehensive conclusions and recommendations on lowering the prevalence of overweight and obesity. However, due to the pressure of schoolwork, Chinese primary and junior high school students did fewer physical activities on a daily basis in the year of this survey and earlier, the physical activity module was not involved in this survey. Despite these facts, the research methods in this paper could still be used by other researchers to study countries or regions with similar sample characteristics and prevalence of overweight and obesity. However, the variable construction method needs to be adjusted appropriately according to the specific sample.

## 5. Conclusions

This paper estimated the possible influential pathway through which students’ subjective dietary knowledge may affect the consumption of SSCBs, thereby affecting the occurrence of overweight and obesity. The estimation results of the hypothetical mediational models show that the mediation effects of the two SSBC consumption indicators were small but significant. In particular, SSCBs consumption frequency had a stronger mediating effect than SSCBs consumption quantity. The heterogeneity effect of mediation by subgroups indicated that the mediation coefficients for girls were more significant and higher in absolute value than those for boys, those for primary school students were more significant and higher in absolute value than those for junior school students, and there was no mediated effect for students with at least one parent with BMI ≥ 30. Considering the heterogeneity between subgroups of mediating effects, public health practitioners and policy makers should adopt a multi-layered nutrition intervention policy to not only strengthen the nutrition education of students, but also to improve the nutrition awareness of parents and school teachers, as well as to strengthen the supervision of children’s consumption of SSBs from the perspective of family and society. First, the education department should institute nutrition and health courses in kindergarten and elementary schools to shape healthy nutrition-conscious eating habits from the early childhood stage. Secondly, for adults, especially parents, the health department should promote healthy eating habits such as reducing sugar intake. Encouraging the whole society to participate in healthy eating actions such as reducing salt, oil, and sugar can create a beneficial environment for controlling children’s sugar intake. Moreover, the government should stipulate prohibiting the excessive promotion of sugar-sweetened beverage products in social welfare institutions dedicated to serving minors and places that mainly provide education, teaching, and activities for minors. Sellers of carbonated beverages should affix standard reminder labels on shelves or counters related to the nutrition of carbonated beverages. The production standards and setting of specifications of relevant reminder slogans should be formulated by the local health department and announced to the public. These steps will make important contributions to reducing the epidemic of overweight and obesity among children and adolescents.

## Figures and Tables

**Table 1:** Sample distribution according to selected personal characteristics.

Characteristic ^1^	Total	Gender		Hukou		Grade			
		Boys	Girls	Urban	Rural	4th	5th	7th	8th
Frequency	3949	1892	2057	1733	1622	1655	804	1044	446
Percentage	100.0%	47.9%	52.1%	51.6%	48.4%	41.9%	20.4%	26.4%	11.3%

^1^ China’s hukou system is the household registration system used in Mainland China. Every citizen is classified as an agricultural or non-agricultural household registration (usually called a rural hukou or an urban hukou) and is further classified by place of birth. Source: Author’s survey.

**Table 2:** Statistics of dependent variables, independent variables, and control variables.

Variable ^1^	Definition	Obs.	Mean	Standard Deviation
***Outcomes***				
BMI–for–age z–score	Body mass index–for–age z–score	3949	0.33	1.33
Overweight	Dummy: =1 if yes (z–score > +1sd); =0 if normal (z–score ≥ −2sd, and z–score ≤ +1sd)	3840	0.32	0.47
Obesity	Dummy: =1 if yes (z–score > +2sd); =0 if normal (z–score ≥ −2sd, and z–score ≤ +1sd)	3130	0.16	0.37
***Dietary Knowledge/SSCBs Consumption***				
Dietary knowledge	Subjective dietary knowledge of student	3949	20.71	3.48
SSCBs_mL	Consumption of SSCBs per day (units: mL/d)	3949	38.09	46.08
SSCBs_freq	Consumption frequency of SSCBs per week (units: drink/w)	3949	1.34	1.55
***Personal/Family Char*** ***acteristics***				
Boy	Dummy: =1 if the student is a boy	3949	0.48	0.50
Agemonth	Age measured by month	3949	140.98	18.79
Preschool	Dummy: =1 if the student had received pre-school education	3949	0.98	0.15
Sibling number	The number of siblings	3949	0.81	0.73
Age_father	Age of father	3949	41.31	5.65
Age_mother	Age of mother	3949	39.09	5.37
Edu_father	Educational years of father	3949	3.54	1.18
Edu_mother	Educational years of mother	3949	3.39	1.27
BMI_father	Body mass index of father	3949	24.18	3.41
BMI_mother	Body mass index of mother	3949	23.06	3.13
Household assets	The household durable asset index	3949	−0.13	0.79

^1^ Source: Authors’ survey.

**Table 3:** BMI–for–age z–score distribution of students by personal/family characteristics in full sample and subgroups.

Variable ^1^	All	Subgroup									Mean Difference				
		Gender		School Type		Only Child		BMI of Parent							
		Boys	Girls	Primary School	Junior High School	Yes	No	Both Normal	Either ≥ 25	Either ≥ 30					
	(1)	(2)	(3)	(4)	(5)	(6)	(7)	(8)	(9)	(10)	(2)–(3)	(4)–(5)	(6)–(7)	(8)–(9)	(8)–(10)
BMI–for–age z–score	0.33	0.51	0.15	0.34	0.31	0.40	0.29	0.13	0.54	0.72	0.36 ***	0.03	0.11 **	−0.41 ***	−0.60 ***
Overweight	0.32	0.40	0.24	0.33	0.29	0.34	0.30	0.26	0.38	0.44	0.16 ***	0.04 **	0.04 **	−0.12 ***	−0.18 ***
Obesity	0.16	0.25	0.09	0.18	0.14	0.19	0.15	0.11	0.22	0.28	0.16 ***	0.04 ***	0.04 ***	−0.11 ***	−0.17 ***

^1^ Robust standard errors in parentheses. *** *p* < 0.01, ** *p* < 0.05, * *p* < 0.1. Source: Authors’ survey.

**Table 4:** Mediation analysis based on standardized regression weights from simultaneous equation modeling with SSCBs_mL as a mediator (for the entire sample).

Variables ^1^	1.		2.		3.	
	SSCBs_mL	BMI–for–Age z–Score	SSCBs_mL	Overweight (=1 if yes)	SSCBs_mL	Obesity (=1 if yes)
DK	−0.776 ***	−0.003	−0.806 ***	0.001	−0.542 **	0.001
	(0.210)	(0.006)	(0.213)	(0.002)	(0.232)	(0.002)
SSCBs_mL	-	0.002	-	0.002 **	-	0.004 ***
	-	(0.003)	-	(0.001)	-	(0.001)
Control variables	Yes	Yes	Yes	Yes	Yes	Yes
Observations	3949	3949	3840	3840	3130	3130
R-squared/Chi2	0.03/102.6	0.06/290.2	0.03/101.8	0.02/279.6	0.03/83.8	−0.14/278.1
Mediated effect (α*β)	−0.002		−0.002 *		−0.002 **	
	(0.002)		(0.001)		(0.001)	
Mediation ratio	Complete		Complete		Complete	

^1^ Robust standard errors in parentheses. *** *p* < 0.01, ** *p* < 0.05, * *p* < 0.1. Source: Authors’ survey.

**Table 5:** Mediation analysis based on standardized regression weights from simultaneous equation modeling with SSCBs_freq as a mediator (for the entire sample).

Variables ^1^	1.		2.		3.	
	SSCBs_freq	BMI–for–Age z–Score	SSCBs_freq	Overweight (=1 if yes)	SSCBs_freq	Obesity (=1 if yes)
DK	−0.040 ***	0.003	−0.040 ***	0.003	−0.035 ***	0.004 *
	(0.007)	(0.007)	(0.007)	(0.002)	(0.008)	(0.002)
SSCBs_freq	-	0.204 ***	-	0.110 ***	-	0.136 ***
	-	(0.079)	-	(0.028)	-	(0.024)
Control variables	Yes	Yes	Yes	Yes	Yes	Yes
Observations	3949	3949	3840	3840	3130	3130
R-squared/Chi2	0.02/97.6	0.02/292.7	0.02/96.4	−0.05/284.8	0.02/72.9	−0.23/285.0
Mediated effect (α*β)	−0.008 **		−0.004 ***		−0.005 ***	
	(0.003)		(0.001)		(0.001)	
Mediation ratio	Complete		Complete		Partial	

^1^ Robust standard errors in parentheses. *** *p* < 0.01, ** *p* < 0.05, * *p* < 0.1. Source: Authors’ survey.

**Table 6:** Mediation analysis based on standardized regression weights from simultaneous equation modeling (for subgroups).

Relationship	Subgroup		Mediator	Direct Effect with Mediator (γ)	Dietary Knowledge on Mediator (α)	Mediator on Health Outcome (β)	Mediated Effect (α*β)	Mediation
**Dietary Knowledge with OverWeight**	**Panel A:** Gender	Boys	SSCBs_mL	−0.003	−0.818 **	−0.002 **	0.002	NA
			SSCBs_freq	−0.003	−0.047 ***	−0.049	0.002	NA
		Girls	SSCBs_mL	0.002	−0.822 ***	0.005 ***	−0.004 **	Complete
			SSCBs_freq	0.003	−0.032 ***	0.149 ***	−0.005 ***	Complete
	**Panel B:** School type	Primary school students	SSCBs_mL	0.007 **	−0.766 ***	0.010 ***	−0.008 ***	Partial
			SSCBs_freq	0.006 **	−0.038 ***	0.182 ***	−0.007 ***	Partial
		Junior school students	SSCBs_mL	−0.005	−0.937 **	−0.001	0.001	NA
			SSCBs_freq	−0.003	−0.044 ***	0.018	−0.001	NA
	**Panel C:** Sibling number	Only-child (no sibling)	SSCBs_mL	−0.004	−1.499 ***	0.003 ***	−0.004 **	Complete
			SSCBs_freq	−0.005	−0.054 ***	0.086 **	−0.005 **	Complete
		At least one sibling	SSCBs_mL	0.003	−0.460 *	0.001	−0.000	NA
			SSCBs_freq	0.006 **	−0.033 ***	0.092 ***	−0.003 **	Partial
	**Panel D:** Parental health status	BMI_father ∈[18.5, 24.9] and BMI_mother ∈[18.5, 24.9]	SSCBs_mL	−0.001	−0.230	0.000	−0.000	NA
			SSCBs_freq	−0.001	−0.032 ***	0.014	−0.000	NA
		BMI_father ≥ 25 or BMI_mother ≥ 25	SSCBs_mL	0.002	−1.282 ***	0.003 **	−0.004 **	Complete
			SSCBs_freq	0.004	−0.041 ***	0.131 ***	−0.005 ***	Complete
		BMI_father ≥ 30 or BMI_mother ≥ 30	SSCBs_mL	0.000	−1.633 *	0.006 ***	−0.009	NA
			SSCBs_freq	0.002	−0.052	0.203 ***	−0.010	NA
Dietary knowledge with Obesity	**Panel A:** Gender	Boys	SSCBs_mL	0.001	−0.505	0.002 *	−0.001	NA
			SSCBs_freq	0.002	−0.038 ***	0.045	−0.002	NA
		Girls	SSCBs_mL	0.000	−0.614 **	0.006 ***	−0.004 **	Complete
			SSCBs_freq	0.001	−0.031 ***	0.149 ***	−0.005 ***	Complete
	**Panel B:** School type	Primary school students	SSCBs_mL	0.003	−0.471 *	0.011 ***	−0.005 *	Complete
			SSCBs_freq	0.005 *	−0.034 ***	0.182 ***	−0.006 ***	Partial
		Junior school students	SSCBs_mL	−0.002	−0.673	0.001	−0.001	NA
			SSCBs_freq	0.002	−0.034 **	0.131 ***	−0.004 **	Complete
	**Panel C:** Sibling number	Only-child	SSCBs_mL	−0.003	−1.472 ***	−0.000	0.000	NA
			SSCBs_freq	−0.002	−0.048 ***	0.031	−0.001	NA
		At least one sibling	SSCBs_mL	0.001	−0.101	0.004 ***	−0.000	NA
			SSCBs_freq	0.005 *	−0.028 ***	0.162 ***	−0.005 **	Partial
	**Panel D:** Parental health status	BMI_father ∈[18.5, 24.9] and BMI_mother ∈[18.5, 24.9]	SSCBs_mL	−0.002	−0.071	0.001	−0.000	NA
			SSCBs_freq	0.001	−0.029 ***	0.076 ***	−0.002 **	Complete
		BMI_father >= 25 or BMI_mother >= 25	SSCBs_mL	0.001	−0.916 ***	0.003 **	−0.003 **	Complete
			SSCBs_freq	0.002	−0.031 ***	0.131 ***	−0.004 **	Complete
		BMI_father >= 30 or BMI_mother >= 30	SSCBs_mL	0.007	−2.149 **	0.006 ***	−0.014 *	Complete
			SSCBs_freq	0.002	−0.043	0.185 ***	0.008	NA

^1^ Robust standard errors in parentheses. *** *p* < 0.01, ** *p* < 0.05, * *p* < 0.1. Source: Authors’ survey. (^2^) NA = Not applicable.

## Data Availability

The data presented in this study are available on request from the corresponding author. The data are not publicly available due to ethical.

## Appendix A

**Table A1:** The subjective dietary knowledge module of the questionnaire for students.

* Ask the Respondent if He or She Strongly Agrees, Somewhat Agrees, Neutral, Somewhat Disagrees or Strongly Disagrees with Each Statement in Item 2 and Record the Answers in Table I5.	
Table I5. Dietary Knowledge	
1	2
Statement Please use 1–5 to describe if you strongly disagree, somewhat disagree, neutral, somewhat agree, or strongly agree with this statement. * Please note that the question is not asking about your actual habits.	1 strongly disagree 2 disagree 3 neutral 4 agree 5 strongly agree 0 unknown
Choosing a diet with a lot of fresh fruits and vegetables is good for one’s health.	_I5-1.
Eating a lot of sugar is good for one’s health.	_I5-2.
Consuming milk and dairy products is good for one’s health	_I5-3.
The heavier one’s body is, the healthier he or she is.	_I5-4.
The development of good eating habits is very beneficial to health	_I5-5.
